# Episodic memory and executive functions in cognitively healthy individuals display distinct neuroanatomical correlates which are differentially modulated by aging

**DOI:** 10.1002/hbm.24306

**Published:** 2018-07-04

**Authors:** Raffaele Cacciaglia, José Luis Molinuevo, Gonzalo Sánchez‐Benavides, Carles Falcón, Nina Gramunt, Anna Brugulat‐Serrat, Oriol Grau, Juan Domingo Gispert, for the ALFA Study, Jordi Camí, Karine Fauria, Marc Suárez‐Calvet, Carolina Minguillón, Gemma Salvadó, Gregory Operto, Marta Crous‐Bou, Albina Polo, Cristina Mustata, Laia Tenas, Paula Marne, Xavi Gotsens, Tania Menchón, Anna Soteras, Laura Hernandez, Ruth Dominguez, Sandra Prades, Maria Pascual, Gema Huesa, Marc Vilanova, Sabrina Segundo, Jordi Huguet

**Affiliations:** ^1^ Barcelonaβeta Brain Research Center, Pasqual Maragall Foundation Barcelona Spain; ^2^ Institut d'Investigacions Biomèdiques August Pi i Sunyer (IDIBAPS) Barcelona Spain; ^3^ CIBER Fragilidad y Envejecimiento Saludable (CIBERFES) Madrid Spain; ^4^ Centro de Investigación Biomédica en Red de Bioingeniería, Biomateriales y Nanomedicina (CIBER‐BBN) Madrid Spain; ^5^ Universitat Pompeu Fabra Barcelona Spain

**Keywords:** aging, default mode network, episodic memory, executive control network, executive functions, voxel based morphometry (VBM)

## Abstract

The neuroanatomical bases of episodic memory (EM) and executive functions (EFs) have been widely addressed in patients with brain damage and in individuals with neurologic disorders. These studies reported that larger brain structures support better outcomes in both cognitive domains, thereby supporting the “bigger is better” account. However, relatively few studies have explored the cerebral morphological properties underlying EM and EFs in cognitively healthy individuals and current findings indicate no unitary theoretical explanation for the structure–function relationship. Moreover, existing studies have typically restricted the analyses to a priori defined regions of interest. Here we conducted unbiased voxel‐wise analysis of the associations between regional gray as well as white matter volumes (GMv; WMv) and performance in both cognitive domains in a sample of 463 cognitively intact individuals. We found that efficiency in EM was predicted by lower GMv in brain areas belonging to the default‐mode network (DMN). By contrast, EFs performance was predicted by larger GMv in a distributed set of regions, which overlapped with the executive control network (ECN). Volume of white matter bundles supporting both cross‐cortical and interhemispheric connections was positively related to processing speed. Furthermore, aging modulated the relationship between regional volumes and cognitive performance in several areas including the hippocampus and frontal cortex. Our data extend the critical role of the DMN and ECN by showing that variability in their morphological properties, and not only their activation patterns, affects EM and EFs, respectively. Moreover, our finding that aging reverts these associations supports previously advanced theories of cognitive neurodevelopment.

## 1 INTRODUCTION

Episodic memory (EM) and executive functions (EFs) are cognitive abilities critical for daily life and display prominent decline in neurological disorders such as Alzheimer's disease (AD) as well as in normal aging (Buckner, 2004). EM refers to the ability of encoding, storing, and consciously recollecting previously learnt events over variable periods ranging from minutes to years. EFs include a variety of abilities that enable goal‐directed behavior through strategy selection, information monitoring, and planning sequencing of actions. Performance in EM has been traditionally related to intact medial temporal lobe (MTL) structures, whereby the hippocampus orchestrates learning and retrieval in conjunction with the entorhinal, perirhinal, and parahippocampal cortices (Dickerson & Eichenbaum, 2010). By contrast, EFs largely rely on the integrity of prefrontal and other frontal regions, and to some extent of parietal cortex (Friedman & Miyake, 2017; Gläscher et al., 2009). Earlier seminal works conducted on patients with focal brain lesions showed that EM and EFs, although functionally related, are in part behaviorally dissociable, that is, patients with selective MTL damage display EFs within normal psychometric ranges (Augustinack et al., 2014; Buckner, 2004; McKenna & Gerhand, 2002; Rosenbaum et al., 2005), while patients suffering from prefrontal insult demonstrate relatively preserved aspects of EM, especially in recognition memory (Milner, Corsi, & Leonard, 1991; Shimamura, Janowsky, & Squire, 1990; Wheeler & Stuss, 2003). Recent functional neuroimaging investigations have confirmed previous lesion studies and additionally revealed the existence of a distributed network subserving both EM and EFs. These studies indicate that, besides the critical role of the hippocampal formation, the network supporting EM encompasses prefrontal and posterior brain areas, including the posterior cingulate cortex (PCC) and angular gyrus, together with activity in content‐specific areas depending on the sensory modality involved during encoding (Rugg & Vilberg, 2013). Regarding EFs, regions other than prefrontal cortex have been documented to display activation for specific tasks related to the executive control, including the insula, the thalamus, and the cerebellar crus (Niendam et al., 2012).

The wealth of functional neuroimaging studies parallels the scarcity of studies on the whole‐brain morphological properties underlying these cognitive faculties. This is despite that regional brain volumes have been shown to predict individual differences in several aspects of human behavior including perception, motor control, and aspects of consciousness (Kanai & Rees, 2011). Structural neuroimaging studies have predominantly investigated cognitive performance in relation to the gray matter volumes in a priori defined regions of interest. These works found discordant evidence on a positive or negative association between hippocampal volume and EM, although a prominent inverse relationship has been reported in younger as opposed to older individuals (see Van Petten (2004) for a review). On the other hand, volumes of prefrontal cortices have more consistently been found to be positively associated to EFs (Yuan & Raz, 2014). Yet, most of the studies have been conducted in individuals with a psychiatric or neurological diagnosis, and less attention has been drawn to the healthy population. However neurologic disorders such as AD, characterized by severe impairment of both EM and EFs, have now been redefined along a disease continuum where the borders between health and pathology appear to be faded (Donohue et al., 2014; Dubois, 2017; Sperling et al., 2011). Thus, understanding the brain morphology supporting memory and executive control in cognitively intact individuals provides valuable information which may represent a reference point to better understand the changes occurring in the preclinical stages of the pathology.

Another important aspect is that both EM and EFs have been shown to rely on processing speed (Salthouse, 2000), a cognitive ability that depends on the integrity of the white matter fiber bundles. To this respect, previous studies reported that reduced processing speed was related to impaired white matter, as indexed by both micro and macrostructural integrity (Borghesani et al., 2013; Kochunov et al., 2010). In another study, Kochunov et al. (2009) further demonstrated a positive association between white matter span and executive control.

In the present study, we aimed to determine the brain structural correlates of EM and EFs in cognitively healthy middle‐aged individuals by analyzing both gray and white matter volumes (GMv; WMv), using an unbiased whole brain voxel‐wise approach. Given the discrepant findings on the brain structural correlates of EM earlier reported, we did not have a directional hypothesis for this cognitive domain. By contrast, we expected to find positive associations between cerebral regional volumes and EFs.

Further, based on prior theories of cognitive neurodevelopment (Van Petten, 2004) we sought to determine whether aging affects the relationship between cognitive performance and regional cerebral volumes.

## 2 METHODS

### 2.1 Study design

We cross‐sectionally evaluated middle‐aged cognitively intact individuals with respect to their performance in episodic memory as well as executive functions, including cognitive processing speed, abstract reasoning, verbal reasoning, and working memory. Further, we tested for significant associations between metrics of cognitive performance and cerebral gray as well as white matter volumes. Finally, to test the hypothesis that aging modifies these associations, we modeled the interaction between age and scores in both cognitive domains of EM and EFs.

### 2.2 Study participants

All subjects were enrolled in the ALFA (ALzheimer and FAmilies) study (ALFA; http://clinicaltrials.gov Identifier: NCT01835717), a large cohort program pointing to the identification of neuroimaging biomarkers of preclinical AD in the general population (Molinuevo et al., 2016). Participants were cognitively healthy with a Clinical Dementia Rate score = 0. Inclusion criteria have been described in detail previously (Molinuevo et al., 2016). From the original ALFA cohort consisting of 2,743 healthy individuals, a sample of subjects was invited to undergo a magnetic resonance imaging (MRI) session, according to their Apolipoprotein E (*APOE*) genotype, as previously described (Cacciaglia et al., in press). Briefly, the subsample for the imaging session was set‐up with the goal of partitioning the genetic variance across five subgroups matched for demographic variables, corresponding to five *APOE* haplotypes (i.e., ε2/ε3, ε2/ε4, ε3/ε3, ε3/ε4, and ε4/ε4). This sampling strategy resulted in 576 study participants, out of which 43 had to be discarded due to either MRI incidental findings or poor image quality. Of the remaining 533 subjects we excluded from the present study all individuals being homozygous for the *APOE‐ε4* risk allele for AD (*N* = 64), as this condition is related to significant brain structural (Cacciaglia et al., in press) and metabolic (Reiman et al., 2005) differences with respect to the rest of the population. Moreover, it is estimated that 98.5% of the entire population entails individuals with either none (noncarriers) or only one copy (heterozygotes) of such risk variant (Qian et al., 2017). Finally, 6 subjects had to be discarded because of unavailable cognitive data, yielding to a final sample of 463 individuals. *APOE* genotyping is described in the Supporting Information.

### 2.3 Image data acquisition and preprocessing

MRI was conducted with a 3T General Electric scanner (GE Discovery MR750 W). Structural 3D high‐resolution T1‐weighted images were collected using a fast spoiled gradient‐echo (FSPGR) sequence implementing the following parameters: voxel size = 1 mm^3^ isotropic, Repetition Time [TR] = 6.16 ms, Echo Time [TE] = 2.33 ms, inversion time [TI] = 450 ms, matrix size = 256 × 256 × 174, and flip angle = 12°. Images were segmented into GM and WM tissue using the new segment function implemented in Statistical Parametric Mapping software (SPM 12, Wellcome Department of Imaging Neuroscience, London, UK), and located into a common space for subsequent normalization using a 9‐affine parameter transformation. Segmented images were then used to generate a reference template object of the sample, which was warped into a standard Montreal Neurological Institute (MNI) space using the high dimensional DARTEL toolbox (Ashburner, 2007). The generated flow fields and normalization parameters were then implemented to normalize the native GM and WM images to the MNI space. In order to preserve the native local amount of GM as well as WM volume, we applied a modulation step, where each voxel signal's intensity was multiplied by the Jacobian determinants derived from the normalization procedure (Good et al., 2001). Quality control of normalization was assured by checking the sample homogeneity with the computational anatomy toolbox (CAT12) (http://dbm.neuro.uni-jena.de/cat/) using nonsmoothed data, which did not return errors in the registration procedure in any subject. Finally, images were spatially smoothed with a 6 mm full‐width at half maximum (FWHM) Gaussian kernel. Total intracranial volume (TIV) was computed by summing the segmented GM, WM, and CSF for each individual.

### 2.4 Neuropsychological evaluation

The neuropsychological assessment took place on average 10.61 months (*SD* = 5.81) before the MRI session. Episodic memory was assessed using the Memory Binding Test (MBT), an instrument that was developed for detecting subtle memory impairment in cognitively intact population (Buschke, 2014). Previous studies have established the ability of the MBT (formerly referred as Memory Capacity Test [MCT]) to discriminate subjects with cerebral Aβ deposition (Papp et al., 2015), to successfully discriminate individuals with mild cognitive impairment (MCI) from normal elderly subjects (Buschke et al., 2017) and also successfully predict the incidence of MCI longitudinally (Mowrey et al., 2016). The MBT uses a controlled learning procedure to ensure that any recall deficit can be attributed to effective memory impairment and not to deficiency in any other cognitive strategies. During administration, the examinee sequentially learns 2 lists of 16 words written in cards, where each card contains 4 words. The lists share semantic categories, which are used both to control the encoding of the words in learning and as cues during cued recall trials. Four main outcomes are produced: total paired recall (TPR), which is the immediate recall of both lists after semantic cueing; total free recall (TFR), for the immediate free recall of both lists; total delayed paired recall (TDPR), for the delayed (30 min after immediate recall) after semantic cueing; and total delayed free recall (TDFR), for the delayed counterpart of the free recall. In this study the Spanish adapted version of the MBT was used (Gramunt et al., 2016). Executive functions were assessed using the Wechsler Adult Intelligence Scale‐Fourth Edition (WAIS‐IV, Wechsler, 2012), which is the most widely used instrument for measuring intelligence. We administered five WAIS subtests. In the Coding test (cognitive processing speed), participants are given keys that match a numeric digit spanning from 1 to 9 with a symbol. The task is to write the correct symbol aside a list of numbers as fast as they can. In the Digit Span (working memory), participants are read a sequence of numbers, that they have to retain the short working memory register and verbalize sorting them in ascending or descending order. Digit Span subtests were studied separately as previously recommended (Colom, Jung, & Haier, 2007) (digit span forward [DSF], digit span backward [DSB], and digit span sequencing [DSS]). In the Matrix Reasoning (nonverbal problem solving) individuals are presented a matrix of abstract geometrical shapes and requested to select out of several possibilities which figure is missing in the sequence. In the Visual Puzzles (visuospatial processing) individuals are presented with a pattern of abstract figures and have to choose three possible parts to make up that pattern. Finally, in the Similarities test (verbal reasoning), participants are presented with two words and asked how they are alike.

### 2.5 Statistical analysis

Analysis of the behavioral data was conducted with the Statistical Package for the Social Sciences (IBM SPSS Statistics, V.21). To assess the impact of demographic factors on EM as well as EFs performance, we performed a univariate analysis of variance (ANOVA) within the general linear model (GLM) where each cognitive outcome was entered as dependent variable separately and age, sex as well as years of education were entered as independent predictors. To determine the structural brain correlates of EM and EFs we performed separate multiple regression models using the general linear model (GLM) in SPM12, where scores of each scale as well as age, sex, years of education, and TIV were included as independent regressors. To investigate the impact of aging in modulating the relationship between GMv as well as WMv and cognitive performance, we performed separate regression analyses, where the interaction term involving age and cognitive score was added in the design matrix. In these latter models, to control for multicollinearity, the regressors encoding cognitive performance were centered at their respective mean values. Furthermore, we analyzed the cerebral morphological correlates in the three different age groups split according to tercile grouping. Results were considered significant if surviving a whole‐brain voxel‐wise statistical threshold of *p* < .001 applying a cluster extent threshold correction of 100 contiguous voxels. This procedure is reliably conservative and further protects against Type I error (Gispert et al., 2015; Wishart et al., 2006). Finally, to reduce dimensionality and to search for common patterns of brain morphology variability associated to EM and EFs, we additionally performed a principal component analysis separately performed for the two cognitive domains and repeated all the above analyses using the extracted principal components as dependent variables (Gaskin & Happell, 2014). To determine the interdependence between EM and EFs, we performed a two‐tailed correlational analysis (Pearson's *r*) between the principal components of the two respective cognitive domains.

## 3 RESULTS

### 3.1 Demographic characteristics and cognitive performance

Table 1 displays demographic characteristics of our sample as well as the group‐mean scores for each cognitive outcome. Table 2 shows the results of the univariate ANOVAs on the predictors of cognitive performance. As expected, there was a significant main effect of age for all cognitive measures, except for a trend in the DSF, indicating that aging was related to worse performance (Supporting Information Figure 1). Female participants performed better than males in EM, with this difference reaching significance in the TFR, TDPR, and TDFR. An opposite pattern was found for EFs, where males significantly outperformed females in all but one (Coding) outcomes. Finally, years of education significantly predicted better performance in all scales. Adding *APOE‐ε4* genotype as covariate in the model as a dichotomous variable (i.e., carriers and noncarriers) did not significantly change the results (Supporting Information Table 1).

**Table 1:** Sample demographic characteristics and cognitive performance stratified by sex

	Whole sample (*N* = 463)		Males (*N* = 188)		Females (*N* = 275)	
	*M*	*SD*	*M*	*SD*	*M*	*SD*
Age^a^	57.18	7.55	57.52	7.588	56.95	7.532
Education^a^	13.68	3.85	14.26	3.361	13.28	3.676
TIV^b^	1,489.99	148.09	1,609.75	116.12	1,408.12	105.8671
MBT‐TPR	24.02	4.55	23.68	4.604	24.25	4.513
MBT‐TFR	16.35	5.16	15.83	4.982	16.7	5.273
MBT‐TDPR	23.74	4.67	23.28	4.607	24.06	4.698
MBT‐TDFR	16.68	5.10	16.13	4.841	17.05	5.251
WAIS‐coding	65.03	15.47	64.94	15.165	65.09	15.706
WAIS‐DSF	8.44	2.09	9.04	2.114	8.04	1.987
WAIS‐DSB	7.93	2.04	8.43	2.186	7.6	1.878
WAIS‐DSS	8.30	2.07	8.81	2.007	7.95	2.048
WAIS‐matrix reasoning	16.31	4.31	17.03	4.245	15.81	4.296
WAIS‐visual puzzles	13.24	4.28	14.71	4.492	12.23	3.833
WAIS‐similarities	22.60	4.73	23.67	4.635	21.86	4.667

TIV = total intracranial volume; MBT = Memory Binding Test; WAIS = Wechsler Adult Intelligence Scale; TPR = total paired recall; TFR = total free recall; TDPR = total delayed paired recall; TDFR = total delayed free recall; DSF = digit span forward; DSB = digit span backwards; DSS = digit span sequencing.

a Indicated in years.

b Indicated in C m^3^.

**Table 2:** Impact of demographic characteristics on cognitive performance

		Age (years)		Sex		Education (years)	
Episodic memory (EM)		*F*	*p*	*F*	*p*	*F*	*p*
	TPR	11.09	<.01	3.10	.08	14.42	<.01
	TFR	31.54	<.01	4.22	.04	9.02	<.01
	TDPR	10.15	<.01	4.92	.03	15.14	<.01
	TDFR	35.96	<.01	5.11	.02	12.98	<.01
							
Executive functions (EFs)							
	Coding	107.9	<.01	0.80	.37	71.58	<.01
	DSF	3.46	.06	22.60	<.01	19.91	<.01
	DSB	22.84	.01	14.94	<.01	26.89	<.01
	DSS	12.77	<.01	16.19	<.01	31.73	<.01
	Matrix reasoning	47.17	<.01	6.06	.01	80.15	<.01
	Visual puzzles	32.44	<.01	38.52	<.01	39.81	<.01
	Similarities	9.31	<.01	10.95	<.01	101.3	<.01

TPR = total paired recall; TFR = total free recall; TDPR = total delayed paired recall; TDFR = total delayed free recall; DSF = digit span forward; DSB = digit span backwards; DSS = digit span sequencing.

### 3.2 Structural brain correlates of cognitive performance

**Figure 1:**
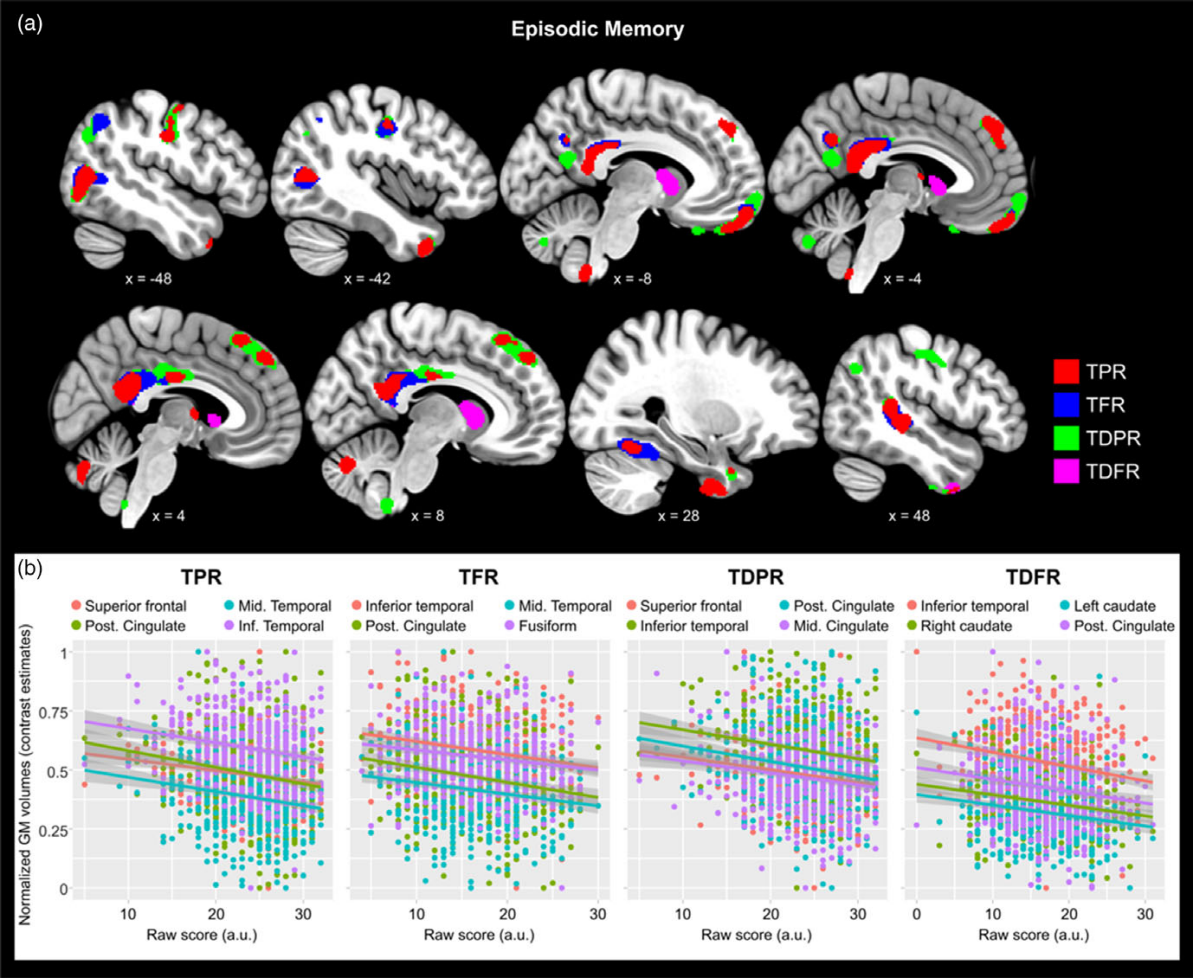
Better performance in episodic memory (EM) was significantly related to lower GMv in several brain areas. (a) Clusters representing the brain regions where lower GMv predicted better EM performance, projected over sagittal slices. Each of the MBT outcomes is coded in different color. Coordinates are provided in the Montreal Neurologic Institute (MNI) standardized space. For visualization purposes the maps are thresholded at uncorrected *p* < .005 (*k* = 100). (b) group scatterplots showing significant negative linear relationships between regional GMv and EM in the different MBT outcomes. GMv values are adjusted for age, sex, years of education, and total intracranial volume (TIV). Shaded areas indicate 90% confidence intervals (TPR = Total paired recall; TFR = Total free recall; TDPR = Total delayed paired recall; TDFR = Total delayed free recall) [Color figure can be viewed at http://wileyonlinelibrary.com]

Better performance in all outcomes of EM was significantly associated to lower gray matter volume in several brain regions (Figure 1; Table 3). Common brain areas explaining variability across all four outcomes of the MBT were the PCC, the left posterior middle temporal cortex, and the right inferior temporal gyrus. In measure of TFR, the negative association with PCC volume additionally survived correction for multiple testing (family‐wise error rate correction [FWE]), on a whole‐brain level. We found no significant positive associations between EM scores and GMv in any brain regions. Additionally, there were no significant relationships between EM and measures of WMv.

**Table 3:** Gray matter volume correlates of cognitive performance in the whole sample

		Brain region	Laterality	*t*‐value*	Cluster size^a^	*x*	*y*	*z*
Episodic memory								
	TPR							
		Frontal sup. medial	R	−4.18	165	5	50	44
		Posterior cingulate	R	−4.08	414	2	−48	21
		Posterior mid. temporal	L	−4.00	205	−50	−71	9
		Inferior temporal	R	−3.93	170	39	0	−50
		Frontal med. orbitalis	L	−3.71	141	−9	60	−23
	TFR							
		Inferior temporal	R	−4.48	207	41	−2	−50
		Posterior cingulate^b^	R	−4.40	756	2	−47	21
		Posterior mid. temporal	L	−3.76	258	−49	−72	9
		Fusiform gyrus	R	−3.57	195	32	−56	−18
	TDPR							
		Frontal med. orbitalis	L	−4.52	460	−9	60	−23
		Frontal sup. medial	R	−4.42	258	0	44	44
		Inferior temporal	R	−4.25	178	41	0	−50
		Posterior mid. temporal	L	−4.14	397	−51	−71	9
		Posterior cingulate	R	−3.76	271	2	−48	23
		Middle cingulate	R	−3.62	170	3	−17	30
	TDFR							
		Inferior temporal	R	−4.70	218	39	2	−51
		Caudate nucleus	R	−4.12	246	15	9	2
		Caudate nucleus	L	−3.87	100	−15	8	3
		Posterior mid. temporal	L	−3.66	130	−48	−71	8
		Frontal med. orbitalis	L	−3.63	101	−11	59	−17
		Posterior cingulate	R	−3.52	155	2	−45	20
Executive functions								
	Coding							
		Thalamus^b^	R	5.12	1,100	0	−14	−2
		Hippocampus	R	4.16	449	14	−15	−20
		Cerebellar crus^b^	L	3.98	1,366	−27	−78	−23
		Cerebellar crus	R	3.56	248	32	−74	−41
								
	DSB							
		Superior frontal	L	4.13	116	−5	41	29
		Cerebellar crus	L	3.91	327	−45	−72	−30
	DSS							
		Anterior mid. temporal	L	4.76	400	−69	−23	−15
		Olfactory nucleus	R	4.08	112	0	8	−11
		Cerebellar crus	L	3.43	168	−41	−75	−35
	Matrix reasoning							
		Caudate nucleus	R	3.95	241	23	18	9
	Visual puzzles							
		Inferior frontal^b^	L	5.50	2,464	−35	30	0
		Inferior parietal^b^	R	5.24	644	27	−45	54
		Inferior temporal^b^	L	5.15	1,010	−35	−6	−36
		Middle frontal	L	4.65	311	−26	38	30
		Middle cingulate	R	4.47	357	15	29	30
		Lingual gyrus^b^	R	4.40	1,251	5	−86	−15
		Cerebellum lob. 8^b^	R	4.38	2,241	12	−54	−47
		Anterior cingulate^b^	L	4.37	1,663	−12	33	24
		Postcentral	R	4.27	461	62	−5	24
		Fusiform gyrus	R	4.24	153	44	−63	−21
		Hippocampus	L	4.12	200	−36	−15	−12
		Superior parietal	L	4.03	112	−14	−69	42
		Postcentral	L	3.97	147	−32	−33	51
		Middle cingulate	L	3.96	372	−8	2	44
		Inferior parietal	L	3.84	137	−42	−35	44
		Inferior temporal	L	3.79	105	−39	−39	−14
		Middle temporal	R	3.78	198	66	−12	−15
		Middle cingulate	R	3.68	180	11	−35	42
		Middle frontal	L	3.67	104	−36	44	21
		Insula	R	3.62	146	36	5	11
		Calcarine	L	3.58	114	−11	−62	11
		Putamen	R	3.47	100	36	−15	−9

TPR = Total paired recall; TFR = Total free recall; TDPR = Total delayed paired recall; TDFR = Total delayed free recall; DSB = digit span backwards; DSS = digit span sequencing.

a Indicated in number of neighboring voxels.

b Survived whole brain family‐wise error rate (FWE) correction for multiple testing. The spatial coordinates refer to the Montreal Neurological Institute (MNI) standardized space.

* Significant at uncorrected *p* < .001 with a cluster extent threshold of *N* = 100 voxels.

**Figure 2:**
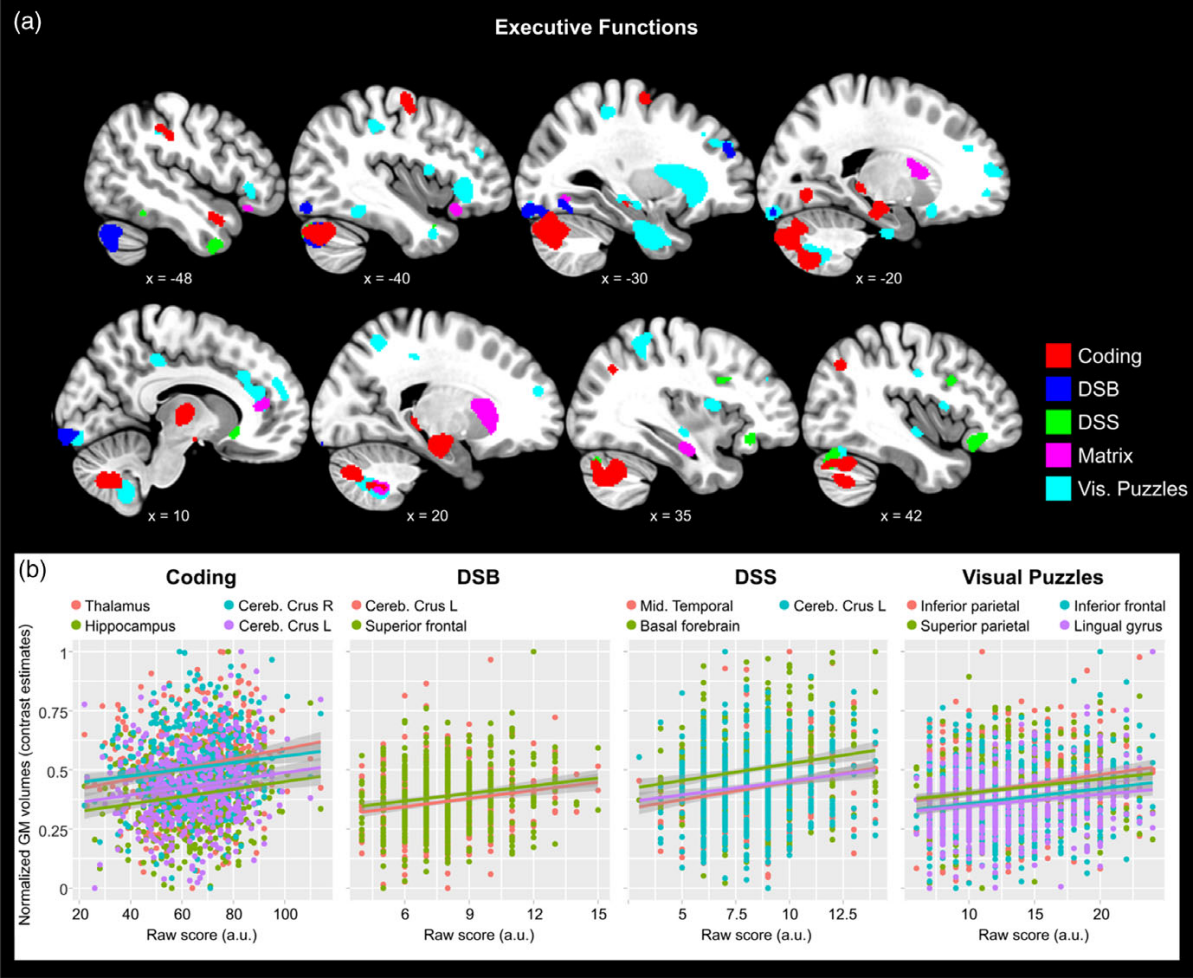
Better performance in executive functions (EFs) was predicted by larger GMv in different brain regions. (a) Clusters representing the brain regions where larger GMv predicted better EFs performance, projected over sagittal slices. Different subscales of the WAIS are color‐coded accordingly. Coordinates are provided in the Montreal Neurologic Institute (MNI) standardized space. For visualization purposes the maps are thresholded at uncorrected *p* < .005 (*k* = 100). (b) Group scatterplots showing significant positive linear relationships between regional GMv and EFs in different WAIS subtests. GMv values are adjusted for age, sex, years of education, and total intracranial volume (TIV). Shaded areas indicate 90% confidence intervals (Coding; DSB = Digit‐span backwards; DSS = digit‐span sequence; Visual Puzzles) [Color figure can be viewed at http://wileyonlinelibrary.com]

**Figure 3:**
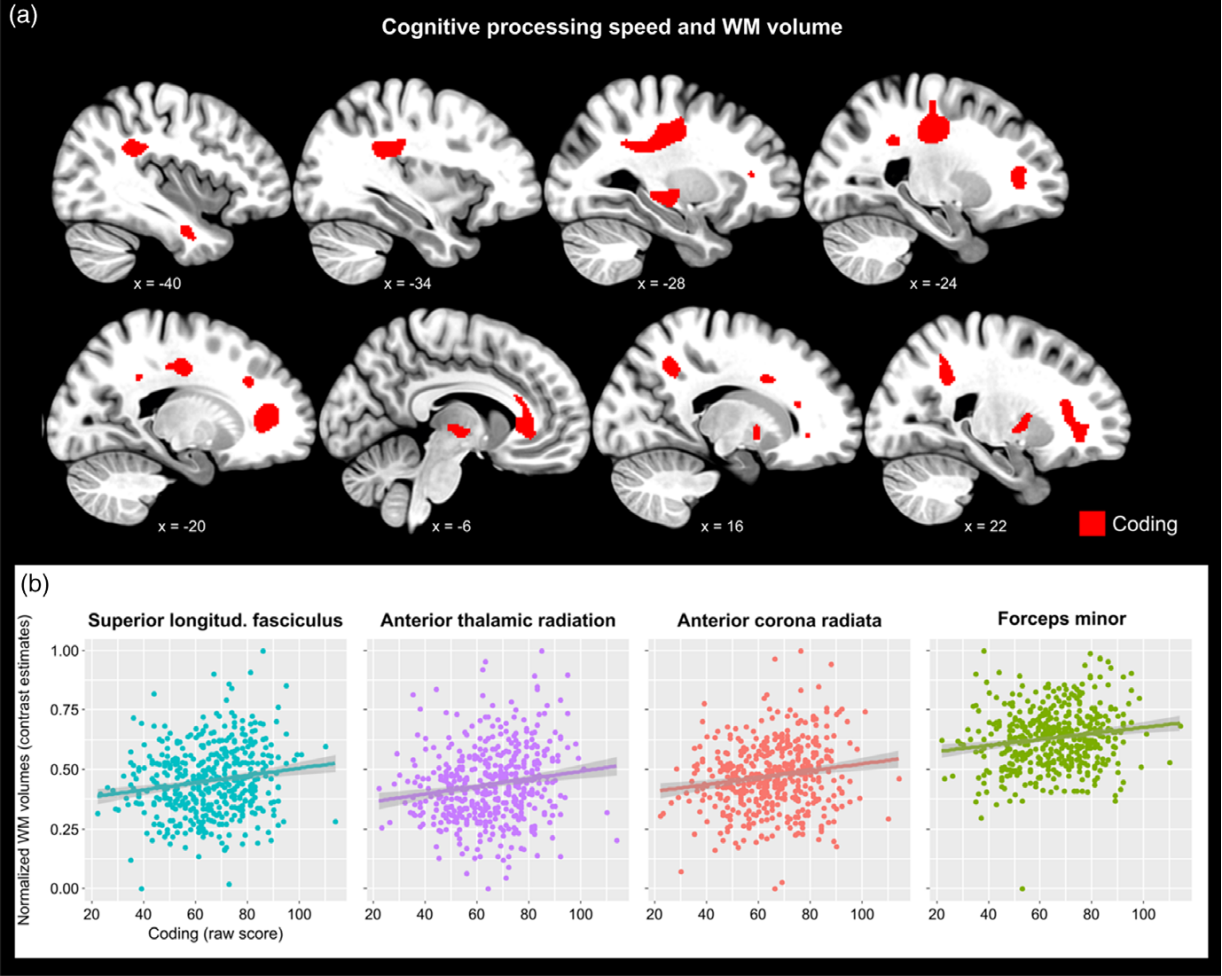
White matter volumes were significantly associated to cognitive processing speed. (a) Volume in several regions of white matter significantly predicted cognitive processing speed, as assessed with the Coding test. (B) Group scatterplots showing significant positive linear relationship between regional white matter volumes and cognitive processing speed in selected regions. Shaded areas indicate 90% confidence intervals [Color figure can be viewed at http://wileyonlinelibrary.com]

By contrast, better performance in EFs was significantly related to larger GMv volumes in frontal and parietal areas as well as in temporal regions and the cerebellum (Figure 2; Table 3). Several areas also survived FWE correction, such as the thalamus and cerebellar crus for the Coding test and the inferior frontal, parietal as well as lingual gyrus and the cerebellum for the Visual Puzzles test (Table 3). We found no significant negative associations between EFs scores and GMv in any brain regions. Cognitive processing speed (Coding test) was significantly associated to WM volumes in several bundles supporting interhemispheric as well as cross‐cortical connections, such as the forceps minor and the superior as well as the inferior longitudinal fasciculi (Figure 3; Table 4). No other measures of executive functions or episodic memory were associated to white matter volume.

**Table 4:** White matter volume correlates of cognitive processing speed (coding test) in the whole sample

		Brain region	Laterality	*t*‐value*	Cluster size^a^	*x*	*y*	*z*
Executive functions								
	Coding							
		Superior longitudinal fasciculus^b^	R	4.33	981	17	−53	42
		Anterior thalamic radiation	L	4.18	353	−3	−11	−3
		Anterior corona radiata^b^	R	4.17	844	32	29	27
		Forceps minor^b^	L	4.14	2,127	−17	39	9
		Inferior longitudinal fasciculus	R	4.12	429	35	−74	6
		Corticospinal tract^b^	L	4.09	2,292	−24	−20	57
		Anterior thalamic radiation^b^	R	4.06	660	8	−15	3
		Inferior fronto‐occipital fasciculus	L	3.93	406	−30	−15	−2
		Optic radiation	L	3.86	176	−39	−8	−27
		Callosal body	R	3.55	154	15	11	35
		Corticospinal tract	R	3.46	208	33	−2	33

a Indicated in number of neighboring voxels.

b Survived whole brain family‐wise error rate (FWE) correction for multiple testing. The spatial coordinates refer to the Montreal Neurological Institute (MNI) standardized space.

* Significant at uncorrected *p* < .001 with a cluster extent threshold of *N* = 100 voxels;

### 3.3 Principal component analysis

**Figure 4:**
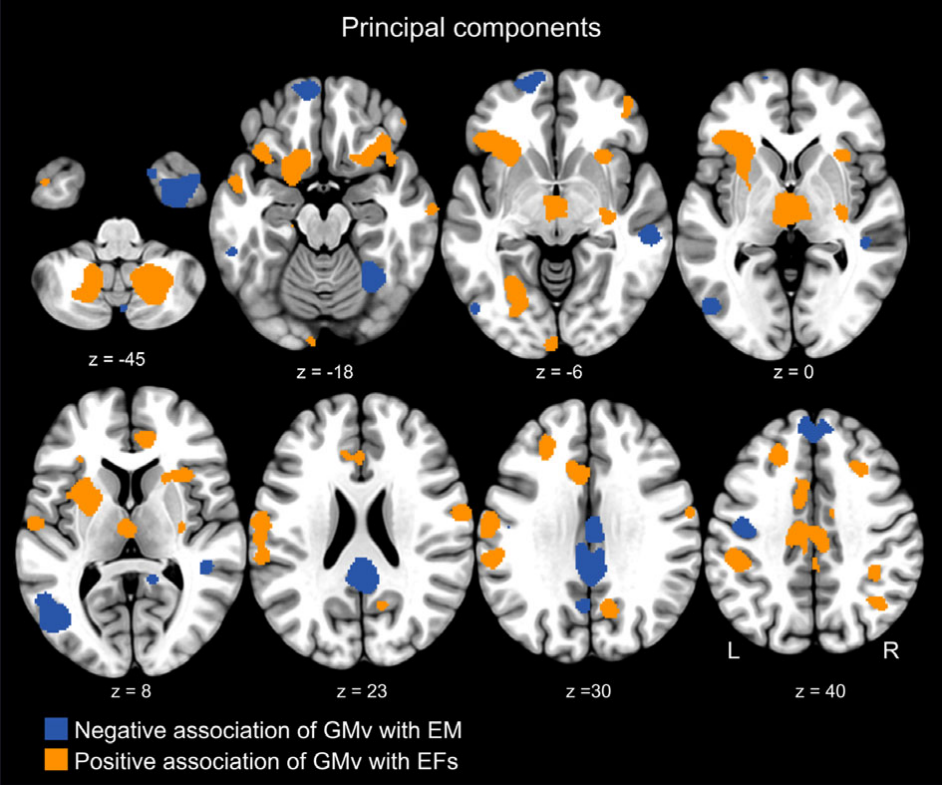
The cerebral morphological correlates underlying EM and EFs were spatially segregated. Superimposition of clusters representing the brain regions computed for the principal components (PCs) relative to EM and EFs. The PC encapsulating EM performance was related to lower GMv in regions such as the posterior cingulate cortex, the medial aspect of the superior frontal cortex, and the posterior middle temporal gyrus. By contrast, the PC representing performance in EFs mapped onto a different set of regions comprising superior as well as inferior frontal regions, inferior parietal, thalamus, cerebellum, and the medial orbitofrontal gyrus. For visualization purposes the maps are thresholded at uncorrected *p* < .005 (*k* = 100) [Color figure can be viewed at http://wileyonlinelibrary.com]

The main principal component for the MBT (PC‐MBT) and the WAIS‐IV (PC‐WAIS) explained 85% and 45.87% of the total variance, respectively. The analysis conducted with the PC‐BMT revealed five brain regions whose GMv was negatively related to EM performance, while PC‐WAIS was positively associated to greater GMv in several brain areas (Supporting Information Table 2). Correlational analysis showed that the two PCs were moderately yet significantly related between each other (*r* = .31; *p* < .01). Overall, the brain structural networks supporting EM and EFs appeared to be spatially segregated and did not show overlapped regions (Figure 4). Analysis of the WM volumes confirmed the lack of associations with EM and corroborated the positive relationship with EFs in a set of white matter bundles (Supporting Information Table 3).

### 3.4 Impact of aging on the relationship between cognitive performance and cerebral volumes

**Figure 5:**
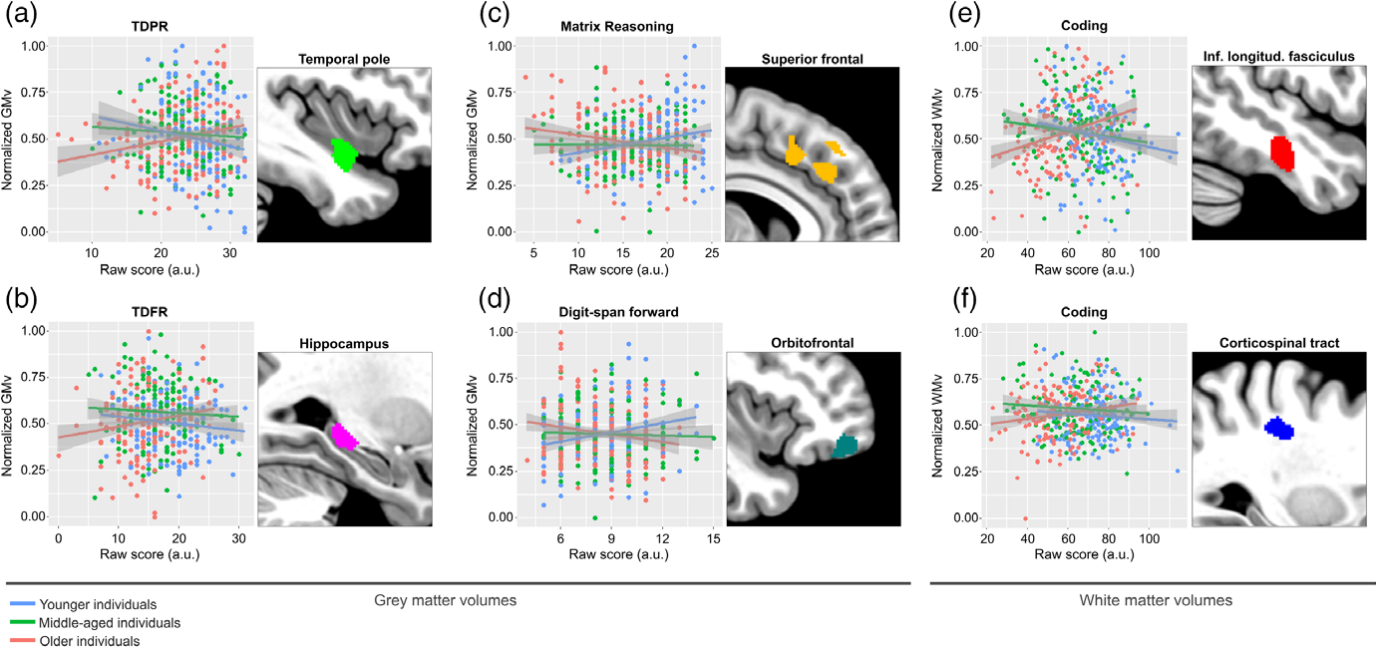
Aging significantly modulated the relationships between cerebral volumes and cognitive performance. Gray matter volumes are plotted against measures of delayed paired recall (a), delayed free recall (b), nonverbal reasoning (c), and working memory (d), in the left temporal pole, left hippocampus, right superior frontal, and right orbitofrontal cortex, respectively. White matter volumes are plotted against measures of cognitive processing speed in the right inferior longitudinal fasciculus (e) and the right dorsal aspect of the corticospinal tract (f), respectively. For visualization purposes the sample was broken down into three different age categories, using tercile grouping. GMv as well as WMv values are adjusted for age, sex, years of education, and total intracranial volume (TIV). Shaded areas in the scatterplots indicate 90% confidence intervals [Color figure can be viewed at http://wileyonlinelibrary.com]

We found significant interactions between age and EM performance in several brain regions. In measures of free recall (i.e., TFR and TDFR) these regions included the hippocampus, the temporal pole, and the posterior cingulum. In measures of paired recall (i.e., TPR and TDPR), the interaction was significant in the orbitofrontal cortex as well as in additional temporal areas (Supporting Information Table 4). The interaction effects in EFs were generally weaker, however, few brain areas survived significance threshold including the orbitofrontal cortex for the DSF, the superior frontal cortex for the Visual Puzzles, and additional brain regions for the Matrix Reasoning (Supporting Information Table 4). Figure 5a–d displays these interaction effects in selected brain regions. When analyzing white matter volumes, we detected significant interactions between age and cognitive processing speed in a set of white matter bundles including the superior as well as the inferior longitudinal fasciculus and the dorsal aspect of the corticospinal tract (Figure 5e–f, Supporting Information Table 5). These results indicate that aging modulates the relationships between brain regional volumes and EM as well as EFs, showing that in older age these associations tend to revert with respect to those in younger ages.

**Figure 6:**
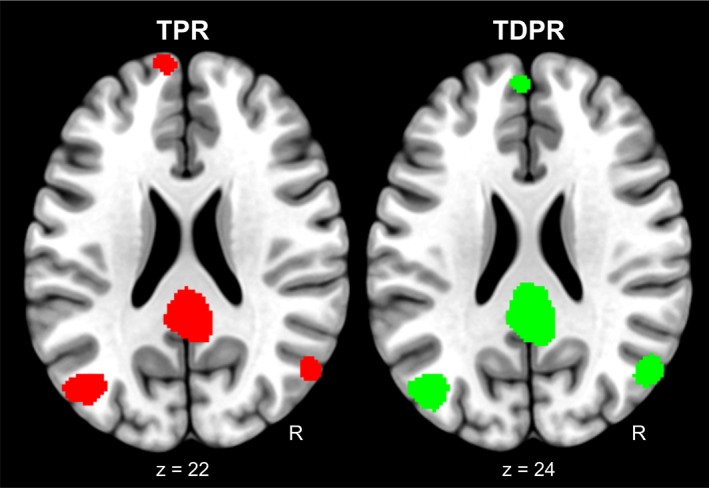
Brain morphometrical correlates of paired recall computed in a subsample of younger individuals. Clusters representing the brain regions where negative associations were found between GMv and TPR and TDPR separately, computed for a subsample of younger individuals. In this subsample, EM performance was significantly predicted by lower GMv in the posterior cingulate cortex (PCC), the bilateral angular gyrus and the medial superior frontal cortex, displaying a set of regions which markedly overlap with the formerly describe default mode network [Color figure can be viewed at http://wileyonlinelibrary.com]

We further analyzed the cerebral GMv correlates in the three different age groups. In younger subjects, the negative correlations with EM were stronger and included the bilateral angular gyri (AG) for measures of paired recall (TPR, right AG: *t* = 3.91; *k* = 65; *p* < .001; left AG: *t* = 4.14; *k* = 252; *p* < .001; TDPR, right AG: *t* = 4.26; *k* = 118; *p* < .001; left AG: *t* = 4.17; *k* = 231; *p* < .001), in addition to medial prefrontal and PCC, yielding a network of regions which is strikingly similar to the previously described default mode network (DMN) (Raichle, 2015) (Figure 6).

## 4 DISCUSSION

We have examined the neuroanatomical patterns underlying episodic memory and executive functions performance in a sample of middle‐aged cognitively healthy individuals using voxel based morphometry (VBM), a methodological approach which does not restrict the investigation in a priori defined regions of interest. Our study was motivated by the limited and heterogeneous findings on the cerebral morphometrical correlates of these cognitive abilities in healthy individuals, which have so far documented both positive and negative associations between GMv and EM and more often a positive relationship with performance in EFs, particularly with the volume of prefrontal regions. Also, since aging has been suggested to modulate the relationship between cerebral morphometry and cognitive performance (Foster et al., 1999; Van Petten, 2004), we looked for interaction effects involving age and our cognitive measures.

First, we found that better EM performance was significantly associated to lower GMv in several brain regions. Common brain areas explaining variability across all four outcomes of the MBT were the PCC, the posterior middle temporal cortex, and the inferior temporal gyrus. This indicates that, regardless of the underlying retrieval processes (i.e.*,* cued vs. free recall) or the laps between encoding and recall (i.e., immediate vs. delayed recall), smaller volume in these three areas predicted better episodic memory performance. In TFR, the negative association with the volume of the PCC additionally survived a whole‐brain FWE correction. Additionally, the volume of medial prefrontal cortex was negatively associated to performance in cued recall (TPR and TDPR). No significant positive associations between regional GMv and EM scores were found.

At first glance, our results might seem surprising given that larger brain regions are typically associated to higher processing capacity and consequently to drive better performance of the functions they mediate (Kanai & Rees, 2011). Also, one would expect to find greater volumes predicting better memory performance, given the close relationship between cognitive decline and brain atrophy observed in AD patients as well as in normal aging (Dickerson & Eichenbaum, 2010). However, at closer inspection, a number of previous studies reported negative associations between regional GMv, mainly for hippocampal volume, and EM in healthy individuals (see Van Petten, 2004 for a review), especially when this was tested on young participants (Chantome et al., 1999; Foster et al., 1999; Pruessner, Pruessner, Hellhammer, Bruce, & Lupien, 2007). These earlier reports suggested that less cerebral regional volumes in younger individuals would be the result of successful neuro‐developmental events, such as synaptic pruning, which optimizes neural computational efficacy. Deficient pruning has been indeed related to several neuropsychiatric conditions which include cognitive disabilities (Paolicelli et al., 2011; Stephan, Barres, & Stevens, 2012). By contrast, reduced GMv in older adults would be the result of neurodegenerative events, and thus greater GMv in advanced age would represent a proxy for available brain reserve, therefore supporting memory performance. Our interaction data showing that aging modulates the relationship between regional GMv and EM support this view. Specifically, for measures of free recall (TFR and TDFR), we observed a significant interaction in the hippocampal volume, with younger individuals displaying a negative relationship while older adults showing a positive association. We also found interaction effects in the same direction for measures of paired recall (TPR and TDPR) in other regions, among which the orbitofrontal cortex and the temporal pole. These results are further supported by earlier findings reporting positive relationships between EM performance and temporal as well as frontal volumes in the older age, as shown in a meta‐analysis (Kaup, Mirzakhanian, Jeste, & Eyler, 2011).

Nevertheless, we should note that other studies found a positive association between hippocampal volume and EM performance in healthy young individuals (e.g., Pohlack et al., 2014). However, the authors computed an index representing the loss in delayed recall with respect the number of learnt words as dependent variable, and did not find significant results when looking at the main outcome of the employed memory test. Such a procedure might have pinpointed a specific cognitive subprocess that may in fact depend on the hippocampal volume.

Critically, the regions we detected in which smaller GMv was associated to better EM performance have been previously shown to be functionally strongly interconnected (Yeo et al., 2011) and more specifically being part of the so‐called task‐negative network, better known as DMN (Raichle, 2015), of which the PCC is considered a central node (Fox et al., 2005). Moreover, when examining the cerebral GMv correlates of EM in younger individuals of our sample, this set of regions included the bilateral angular gyrus, which is also a prominent structure within the DMN. The DMN mediates internal cognitive processes such as self‐referenced thoughts (Davey, Pujol, & Harrison, 2016) and introspective accuracy (Fleming, Weil, Nagy, Dolan, & Rees, 2010). Importantly, it has been shown that deactivation during encoding in the DMN regions, including posterior middle temporal cortices, predicted successful retrieval for the studied items (Chai, Ofen, Gabrieli, & Whitfield‐Gabrieli, 2014; Daselaar, Prince, & Cabeza, 2004; Kim, Daselaar, & Cabeza, 2010), that is, the ability to suppress their response during active memory tasks constitutes a prerequisite for efficient recall. Thus, our findings extend previous knowledge on the critical role for the DMN in episodic memory to the structural level, by showing that also a reduced GM volume, and not just reduced activity, in these areas is associated to efficient episodic recall. This interpretation is further supported by previous studies showing a high degree of shared covariance among the nodes of the DMN in terms of their GM volumetric values (Khalsa, Mayhew, Chechlacz, Bagary, & Bagshaw, 2014; Luo et al., 2012), thus linking cerebral function and structure.

One area that we found to predict delayed free recall, but not any other MBT outcome, is the bilateral caudate nucleus. The caudate is involved in forming stimulus‐response associations in learning tasks (Chiu, Jiang, & Egner, 2017), a process that is relevant for the binding memory process underlying the MBT. Although this region does not belong to the DMN, it has been proposed that dopaminergic signaling and the degree of functional coupling of the caudate with MTL structures is associated with episodic memory (Nyberg et al., 2016). Moreover, we have previously shown that the morphology of the caudate nucleus is sensitive to the *APOE*‐ε4 genotype, displaying a gene dose‐dependent volumetric reduction in cognitively healthy people (Cacciaglia et al., in press). Therefore, the caudate nucleus is a candidate brain structure where the earliest signs of structural alterations in the AD continuum may appear, and its structural properties may be important in modulating episodic memory.

Unlike the findings for EM, we observed that better performance in EFs was predicted by larger GMv in several brain areas. These included the thalamus, the anterior hippocampus and the bilateral cerebellar crus (Coding), medial superior frontal and cerebellar crus (DSB), middle temporal and olfactory nucleus (DSS), as well as a number of cortical regions comprising frontal and parietal areas (Visual Puzzles). Most of these regions have been previously identified as part of the executive control network (ECN), such as the medial portion of the superior frontal, superior and inferior parietal (Seeley et al., 2007), as well as the cerebellar crus (Habas et al., 2009) and the thalamus (Marzinzik et al., 2008). In line with our data, one study using VBM reported that the volume of the cerebellar crus was associated to performance in the DSB in healthy individuals (Ruscheweyh et al., 2013). Additionally, the association we found between DSS performance—a proxy for working memory—and the volume of the olfactory nucleus parallels recent findings on the importance of olfaction in executive functions (Fagundo et al., 2015). The lack of significant associations between GMv and the Similarities test as well the DSF may be due to the fact that, among the scales employed in this study, those are the ones requiring the least cognitive effort.

When examining WM volumes, no significant relationships emerged with EM. We did, however, find a positive association between cognitive processing speed and the volumes in several WM bundles underlying cross‐cortical as well as interhemispheric connections. Significant clusters included the superior longitudinal fasciculus (SLF), the anterior thalamic radiation (ATR), as well as the forceps minor and the dorsal aspect of corticospinal tract (Figure 3). For the SLF, the local maxima peaked around an area that corresponds to its second subdivision (SLF‐II), which connects the angular gyrus to the parieto‐occipital regions supporting visual awareness and maintenance of attention (Schmahmann, Smith, Eichler, & Filley, 2008). The ATR projects to medial prefrontal areas from the anterior thalamic nucleus, which in turn receives afferences from the hippocampus (Mamah et al., 2010). Hence, this finding complements our data on GMv indicating that cognitive processing speed relies on the integrity of a circuit encompassing the hippocampus, the anterior thalamus, and medial prefrontal regions, besides parieto‐occipital loops. Furthermore, we showed that aging modified the associations between cognitive processing speed and the volume in a set of white matter fiber bundles. These included the bilateral inferior longitudinal fasciculus and the dorsal aspect of the corticospinal tract, which are subject to a late myelination process compared to large sensory or midbrain pathways (Lu et al., 2013). There is evidence that late‐myelinating white matter is more susceptible to the age‐related degeneration (Bartzokis, 2004), thus our data may capture this process by showing that older individuals rely more on the integrity of such incipiently deteriorating WM tracts. Overall, our results are well in line with earlier reports on the role of white matter integrity in cognitive processing speed (Kochunov et al., 2010) and more in general executive functions (Borghesani et al., 2013; Kochunov et al., 2009), and further extend previous findings to the macrostructural properties of white matter fiber bundles.

The regions supporting EFs performance were spatially segregated from those subserving EM (Figure 3), despite a moderate yet significant correlation between the respective principal components of each cognitive domain. This topographic segregation represents a brain morphometrical account for the behavioral dissociation already documented in patients with focal brain damage, where aspects of EM were spared in individuals with frontal lesions, while executive control was preserved in people with MTL lesions (Augustinack et al., 2014; Buckner, 2004; McKenna & Gerhand, 2002; Milner et al., 1991; Rosenbaum et al., 2005; Shimamura et al., 1990; Wheeler & Stuss, 2003). Our results show that the cerebral morphological correlates of EFs consistently included positive associations, in striking contrast to the findings observed for EM, where only negative associations were found. This divergence is reconciled within the theories which place cognitive functions along the axis of “perceptual‐conceptual” processing (Cabeza & Moscovitch, 2013). EM tasks can be conceived as “conceptual” processes that operate on internal source of information, especially in recollection‐based retrieval (Li, Mao, Wang, & Guo, 2017). This holds particularly for the MBT which, unlike other episodic memory tests, requires the subjects to learn a relatively high number of semantic categories across the studied items, and thus might partially engage a semantic memory system that involves conceptual manipulation of information (Binder, Desai, Graves, & Conant, 2009). By contrast, EFs resembles a “perceptual” type of process which operates on external source of information derived from immediate, ongoing sensory and motor processes. In this view, our results parallel earlier functional neuroimaging data which pointed out that while conceptual processing engages the so called “task‐negative” network (e.g., deactivation of the DMN), perceptual manipulations involves “task‐positive” systems, such as activation of the executive‐control network (Kim et al., 2010; Murphy et al., 2018).

We also found significant interactions for EFs, indicating an opposite modulatory effect for age with respect to EM. More precisely, younger individuals exhibited a positive association while in older ones this relationship tended to revert for some regional GMv including orbitofrontal cortex (Coding), superior frontal (Visual Puzzles), and the medial aspect of the frontal gyrus (Matrix Reasoning). Interestingly, the only two studies reporting negative associations between measures of EFs and prefrontal volumes in cognitive healthy individuals were conducted in older adults, which supports our interaction data (Duarte et al., 2006; Salat, Kaye, & Janowsky, 2002). Overall, our interaction results indicate that aging reverts the association between regional GMv and cognitive performance in both domains of EM and EFs. One mechanism underlying this differential effect may be due to the distinct lifetime trajectories of GMv loss in different brain structures (Fjell et al., 2009; Smith, Chebrolu, Wekstein, Schmitt, & Markesbery, 2007). Frontal areas are subject to thinning in a higher degree than midline cortical areas where we found the effect for EM, and therefore the brain reserve in these regions would accumulate earlier in life. This, together with the development of complex compensatory neuroplastic events occurring during aging, may explain the opposite role of age in modulating the relationship between GMv and performance in the two cognitive domains. However, the cross‐sectional nature of our study prevents us to determine the trajectories of cognitive scores as well as their underlying neuroimaging correlates. Further longitudinal studies are required to disentangle the precise modulatory role of aging in reverting these relationships in resilient versus vulnerable cognitive aging. Likewise, future studies shall take into account the modulatory role of the *APOE‐ε4* allelic load, which has been previously associated to deficient hippocampal pruning (Chung et al., 2016) and significant cognitive decline assessed longitudinally (Caselli et al., 2009).

Another limitation of our study lays in our univariate statistical approach. Additional multivariate statistical approaches, such as analysis of independent components of GMv variability, shall clarify patterns of cerebral structural covariance underlying cognitive performance.

Taken together, our results show that EM and EFs rely on distinct brain neuroanatomical patterns that closely resemble the DMN and the ECN, respectively. The opposite direction of the observed relationships with regional GMv underscores that EM and EFs belong to two different global cognitive processes. Finally we showed that aging differentially modulate these associations, exerting opposite modulatory roles in the relationship between regional GMv and the two cognitive domains.

## CONFLICT OF INTEREST

None of the authors has any potential conflict of interest related to this manuscript.

## Supporting information

Supplementary Materials
