# Supplementary material for: Episodic memory and executive functions in cognitively healthy individuals display distinct neuroanatomical correlates which are differentially modulated by aging
Source: Hum Brain Mapp. 2018 Jul 4;39(11):4565–79. doi: 10.1002/hbm.24306 (PMC6220988; doi:10.1002/hbm.24306)
Supplement: Supplementary file 1 — Supplementary Materials [file HBM-39-4565-s001.docx]

**Title:** Episodic memory and executive functions in cognitively healthy individuals display distinct neuroanatomical correlates which are differentially modulated by aging

**Supplementary Materials**

***APOE genotyping***

Total DNA was obtained from the blood cellular fraction by proteinase K digestion followed by alcohol precipitation. Samples were genotyped for two single nucleotide polymorphisms (SNPs), rs429358 and rs7412, determining the possible *APOE* isoforms: ε1, rs429358 (C) + rs7412 (T); ε2, rs429358 (T) + rs7412 (T); ε3, rs429358 (T) + rs7412 (C); and ε4, rs429358 (C) + rs7412 (C). Of the 463 participants, 162 were ε3/ε4 carriers, 149 were homozygous for the ε3 allele, 104 were ε2/ε3 carriers, 42 were ε2/ε4, and 6 were ε2/ε2 carriers. The allele frequencies were in Hardy-Weinberg equilibrium.

**Supplementary Table 1 – Cognitive performance in the whole sample (covarying for *APOE-ε4* genotype)**

|  |  |  | **Age (y)** | | |  | **Sex** | | |  | **Education (y)** | | |  | ***APOE*-*ε4* carrier*** | | |
| --- | --- | --- | --- | --- | --- | --- | --- | --- | --- | --- | --- | --- | --- | --- | --- | --- | --- |
| **EM** |  |  | ***F*** |  | **P** |  | ***F*** |  | **P** |  | ***F*** |  | **P** |  | ***F*** |  | **P** |
|  | TPR |  | 11.23 |  | <0.01 |  | 3.37 |  | 0.07 |  | 14.48 |  | <0.01 |  | 0.93 |  | 0.33 |
|  | TFR |  | 31.46 |  | <0.01 |  | 4.18 |  | 0.04 |  | 9.01 |  | <0.01 |  | 0.01 |  | 0.98 |
|  | TDPR |  | 10.27 |  | <0.01 |  | 5.25 |  | 0.02 |  | 15.20 |  | <0.01 |  | 0.83 |  | 0.36 |
|  | TDFR |  | 36.21 |  | <0.01 |  | 5.40 |  | 0.02 |  | 13.04 |  | <0.01 |  | 1.01 |  | 0.31 |
|  |  |  |  |  |  |  |  |  |  |  |  |  |  |  |  |  |  |
| **EFs** |  |  |  |  |  |  |  |  |  |  |  |  |  |  |  |  |  |
|  | Coding |  | 107.4 |  | <0.01 |  | 0.67 |  | 0.41 |  | 71.41 |  | <0.01 |  | 0.57 |  | 0.44 |
|  | DSF |  | 3.54 |  | 0.06 |  | 21.55 |  | <0.01 |  | 19.99 |  | <0.01 |  | 1.10 |  | 0.29 |
|  | DSB |  | 6.13 |  | 0.01 |  | 15.60 |  | <0.01 |  | 26.86 |  | <0.01 |  | 0.08 |  | 0.77 |
|  | DSS |  | 12.69 |  | <0.01 |  | 16.28 |  | <0.01 |  | 31.63 |  | <0.01 |  | 0.12 |  | 0.72 |
|  | Matrices |  | 31.19 |  | <0.01 |  | 39.14 |  | <0.01 |  | 39.69 |  | <0.01 |  | 0.73 |  | 0.39 |
|  | VPs |  | 46.98 |  | <0.01 |  | 6.10 |  | 0.01 |  | 79.94 |  | <0.01 |  | 0.05 |  | 0.81 |
|  | Similarities |  | 9.39 |  | <0.01 |  | 10.44 |  | <0.01 |  | 101.3 |  | <0.01 |  | 0.49 |  | 0.48 |

*Coded in two categories, Non-Carriers and Heterozygotes; EM: Episodic Memory; EFs: Executive Functions; TPR: Total paired recall; TFR: Total free recall; TDPR: Total delayed paired recall; TDFR: Total delayed free recall; DSF: Digit Span Forward; DSB: Digit Span Backwards; DSS: Digit Span Sequencing; VPs: Visual Puzzles

**Supplementary Table 2 – Grey matter volumetric correlates of cognitive performance assessed for the Principal Components (PCs)**

|  |  |  | **Brain region** |  | **Laterality** |  | ***t*-value*** |  | **Cluster size**** |  | **x** |  | **y** |  | **z** |
| --- | --- | --- | --- | --- | --- | --- | --- | --- | --- | --- | --- | --- | --- | --- | --- |
| **Episodic Memory** |  |  |  |  |  |  |  |  |  |  |  |  |  |  |  |
|  | **PC #1 (MBT)** |  |  |  |  |  |  |  |  |  |  |  |  |  |  |
|  |  |  | Inferior temporal |  | R |  | -4.68 |  | 246 |  | 39 |  | 0 |  | -50 |
|  |  |  | Posterior cingulate^●^ |  | R |  | -4.20 |  | 661 |  | 2 |  | -47 |  | 21 |
|  |  |  | Posterior middle temporal |  | L |  | -4.18 |  | 325 |  | -50 |  | -71 |  | 9 |
|  |  |  | Frontal Sup. Medial |  | R |  | -4.10 |  | 112 |  | 0 |  | 44 |  | 44 |
|  |  |  | Frontal Med. Orbitalis |  | L |  | -4.01 |  | 252 |  | -9 |  | 60 |  | -23 |
| **Executive Functions** |  |  |  |  |  |  |  |  |  |  |  |  |  |  |  |
|  | **PC #1 (WAIS)** |  |  |  |  |  |  |  |  |  |  |  |  |  |  |
|  |  |  | Cerebellum Lob. 8^●^ |  | R |  | 4.83 |  | 976 |  | 21 |  | -56 |  | -47 |
|  |  |  | Inferior frontal^●^ |  | L |  | 4.38 |  | 619 |  | -38 |  | 29 |  | -2 |
|  |  |  | Inferior parietal |  | L |  | 4.37 |  | 206 |  | -47 |  | -36 |  | 39 |
|  |  |  | Orbitofrontal medial |  | L |  | 4.22 |  | 236 |  | -17 |  | 18 |  | -18 |
|  |  |  | Thalamus |  | L |  | 4.08 |  | 320 |  | -6 |  | -20 |  | -5 |
|  |  |  | Superior frontal |  | L |  | 4.04 |  | 164 |  | -27 |  | 30 |  | 33 |
|  |  |  | Postcentral |  | L |  | 4.04 |  | 314 |  | -62 |  | -11 |  | 27 |
|  |  |  | Insula |  | R |  | 3.99 |  | 165 |  | 29 |  | 18 |  | -9 |
|  |  |  | Inferior temporal |  | L |  | 3.91 |  | 198 |  | -35 |  | -5 |  | -36 |
|  |  |  | Postcentral |  | R |  | 3.89 |  | 102 |  | 63 |  | -5 |  | 24 |
|  |  |  | Fusiform |  | L |  | 3.87 |  | 287 |  | -27 |  | -65 |  | -11 |
|  |  |  | Middle cingulate |  | L |  | 3.82 |  | 123 |  | -8 |  | -15 |  | 42 |
|  |  |  | Putamen |  | L |  | 3.58 |  | 103 |  | -30 |  | -2 |  | 12 |

*Significant at uncorrected p<0.001 with a cluster extent threshold of N = 100 voxels; **indicated in number of neighboring voxels

^●^Survived whole brain family-wise error rate (FWE) correction for multiple testing. Spatial coordinates are provided in the Montreal Neurological Institute (MNI) standardized space.

**Supplementary Table 3 – White matter volumetric correlates of the first principal component assessed for the WAIS scale**

|  |  |  | **WM bundle** |  | **Laterality** |  | ***t*-value*** |  | **Cluster size**** |  | **x** |  | **y** |  | **z** |
| --- | --- | --- | --- | --- | --- | --- | --- | --- | --- | --- | --- | --- | --- | --- | --- |
| **EFs** |  |  |  |  |  |  |  |  |  |  |  |  |  |  |  |
|  | **PC #1 (WAIS)** |  |  |  |  |  |  |  |  |  |  |  |  |  |  |
|  |  |  | Inferior fronto-occipital fasciculus^●^ |  | L |  | 5.13 |  | 712 |  | -39 |  | -8 |  | -29 |
|  |  |  | Anterior thalamic radiation^●^ |  | L |  | 4.35 |  | 1622 |  | -14 |  | 12 |  | 20 |
|  |  |  | Callosal body |  | R |  | 3.80 |  | 107 |  | 11 |  | -15 |  | 36 |
|  |  |  | Superior longitudinal fasciculus |  | L |  | 3.49 |  | 161 |  | -38 |  | -42 |  | 29 |
|  |  |  | Forceps minor |  | R |  | 3.42 |  | 290 |  | 14 |  | 30 |  | 17 |

EFs: Executive Functions.

*Significant at uncorrected p<0.001 with a cluster extent threshold of N = 100 voxels; **indicated in number of neighboring voxels

^●^Survived whole brain family-wise error rate (FWE) correction for multiple testing. Spatial coordinates are provided in the Montreal Neurological Institute (MNI) standardized space.

**Supplementary Table 4 – Aging modulated the relationships between cognitive performance and GMv in several brain areas**

|  |  |  | **Brain region** |  | **Laterality** |  | ***t*-value*** |  | **Cluster size**** |  | **x** |  | **y** |  | **z** |
| --- | --- | --- | --- | --- | --- | --- | --- | --- | --- | --- | --- | --- | --- | --- | --- |
| **Episodic Memory** |  |  |  |  |  |  |  |  |  |  |  |  |  |  |  |
|  | **TPR** |  |  |  |  |  |  |  |  |  |  |  |  |  |  |
|  |  |  | Temporal pole |  | R |  | 4.43 |  | 116 |  | 45 |  | 26 |  | -20 |
|  | **TFR** |  |  |  |  |  |  |  |  |  |  |  |  |  |  |
|  |  |  | Supramarginal^●^ |  | R |  | 4.22 |  | 491 |  | 47 |  | -32 |  | 27 |
|  |  |  | Temporal pole |  | L |  | 3.90 |  | 115 |  | -42 |  | -3 |  | -21 |
|  |  |  | Hippocampus |  | L |  | 3.87 |  | 101 |  | -27 |  | -32 |  | -6 |
|  | **TDPR** |  |  |  |  |  |  |  |  |  |  |  |  |  |  |
|  |  |  | Temporal pole |  | L |  | 4.26 |  | 262 |  | -42 |  | 0 |  | -14 |
|  |  |  | Posterior middle temporal |  | R |  | 4.08 |  | 189 |  | 45 |  | -63 |  | 12 |
|  |  |  | Temporal pole |  | L |  | 3.84 |  | 143 |  | -30 |  | 8 |  | -35 |
|  | **TDFR** |  |  |  |  |  |  |  |  |  |  |  |  |  |  |
|  |  |  | Temporal pole^●^ |  | L |  | 4.69 |  | 884 |  | -30 |  | 9 |  | -32 |
|  |  |  | Superior temporal^●^ |  | R |  | 4.41 |  | 566 |  | 44 |  | -18 |  | -5 |
|  |  |  | Hippocampus |  | L |  | 4.23 |  | 263 |  | -29 |  | -30 |  | -6 |
|  |  |  | Rectus |  | L |  | 4.20 |  | 423 |  | -17 |  | 23 |  | -15 |
|  |  |  | Inferior frontal |  | R |  | 4.04 |  | 103 |  | 54 |  | 33 |  | -11 |
|  |  |  | PCC |  | R |  | 3.97 |  | 163 |  | 0 |  | -33 |  | 29 |
|  |  |  | Middle temporal |  | R |  | 3.68 |  | 126 |  | 62 |  | -35 |  | 2 |
|  |  |  | Supramarginal |  | R |  | 3.67 |  | 125 |  | 47 |  | -32 |  | 27 |
|  |  |  | Cerebellum Lob. 8 |  | R |  | 3.62 |  | 117 |  | 15 |  | -62 |  | -42 |
|  |  |  | Rectus |  | R |  | 3.58 |  | 107 |  | 17 |  | 17 |  | -17 |
|  | **PC #1 (MBT)** |  |  |  |  |  |  |  |  |  |  |  |  |  |  |
|  |  |  | Temporal pole |  | L |  | 4.14 |  | 197 |  | -42 |  | -3 |  | -21 |
|  |  |  | Supramarginal |  | R |  | 3.98 |  | 175 |  | 47 |  | -32 |  | 27 |
|  |  |  | Temporal pole |  | L |  | 3.96 |  | 175 |  | -30 |  | 9 |  | -32 |
|  |  |  | Hippocampus |  | L |  | 3.87 |  | 108 |  | -29 |  | -30 |  | -6 |
|  |  |  | Superior temporal |  | R |  | 3.65 |  | 121 |  | 48 |  | -27 |  | 5 |
| **Executive Functions** |  |  |  |  |  |  |  |  |  |  |  |  |  |  |  |
|  | **Digit Span FW** |  |  |  |  |  |  |  |  |  |  |  |  |  |  |
|  |  |  | Orbitofrontal |  | R |  | -4.23 |  | 208 |  | 38 |  | 51 |  | -18 |
|  | **Visual Puzzles** |  |  |  |  |  |  |  |  |  |  |  |  |  |  |
|  |  |  | Superior frontal |  | L |  | -4.12 |  | 140 |  | -18 |  | 57 |  | 8 |
|  | **Matrix Reasoning** |  |  |  |  |  |  |  |  |  |  |  |  |  |  |
|  |  |  | Insula^●^ |  | R |  | -4.78 |  | 477 |  | 41 |  | -5 |  | 5 |
|  |  |  | Sup. Frontal medial |  | R |  | -4.15 |  | 101 |  | 3 |  | 38 |  | 35 |
|  |  |  | Precuneus |  | R |  | -4.10 |  | 129 |  | 9 |  | -66 |  | 32 |
|  |  |  | Orbitofrontal |  | R |  | -4.08 |  | 149 |  | 35 |  | 23 |  | -14 |
|  |  |  | Superior frontal |  | L |  | -3.97 |  | 225 |  | -27 |  | 57 |  | 6 |
|  | **PC #1 (WAIS)** |  |  |  |  |  |  |  |  |  |  |  |  |  |  |
|  |  |  | Insula |  | R |  | -4.50 |  | 182 |  | 32 |  | 3 |  | 15 |

*Significant at uncorrected p<0.001 with a cluster extent threshold of N = 100 voxels.

**indicated in number of neighboring voxels. ^●^Survived whole brain family-wise error rate (FWE) correction for multiple testing.

TPR: Total paired recall; TFR: Total free recall; TDPR: Total delayed paired recall; TDFR: Total delayed free recall; PC: Principal component

The spatial coordinates refer to the Montreal Neurological Institute (MNI) standardized space.

**Supplementary Table 5 – Aging modulated the relationships between cognitive processing speed and white matter volumes**

|  |  |  | **White matter bundle** |  | **Laterality** |  | ***t*-value*** |  | **Cluster size**** |  | **x** |  | **y** |  | **z** |
| --- | --- | --- | --- | --- | --- | --- | --- | --- | --- | --- | --- | --- | --- | --- | --- |
| **Executive functions** |  |  |  |  |  |  |  |  |  |  |  |  |  |  |  |
|  | **Coding** |  |  |  |  |  |  |  |  |  |  |  |  |  |  |
|  |  |  | Inferior longitudinal fasciculus^●^ |  | R |  | 4.77 |  | 143 |  | 48 |  | -51 |  | -9 |
|  |  |  | Inferior longitudinal fasciculus |  | L |  | 4.48 |  | 530 |  | -50 |  | -23 |  | -18 |
|  |  |  | Corticospinal tract^●^ |  | R |  | 4.12 |  | 1272 |  | 27 |  | -23 |  | 48 |
|  |  |  | Forceps major |  | L |  | 3.78 |  | 152 |  | -21 |  | -72 |  | 20 |

*Significant at uncorrected p<0.001 with a cluster extent threshold of N = 100 voxels; **indicated in number of contiguous voxels

^●^Survived whole brain family-wise error rate (FWE) correction for multiple testing. The spatial coordinates refer to the Montreal Neurological Institute (MNI) standardized space.


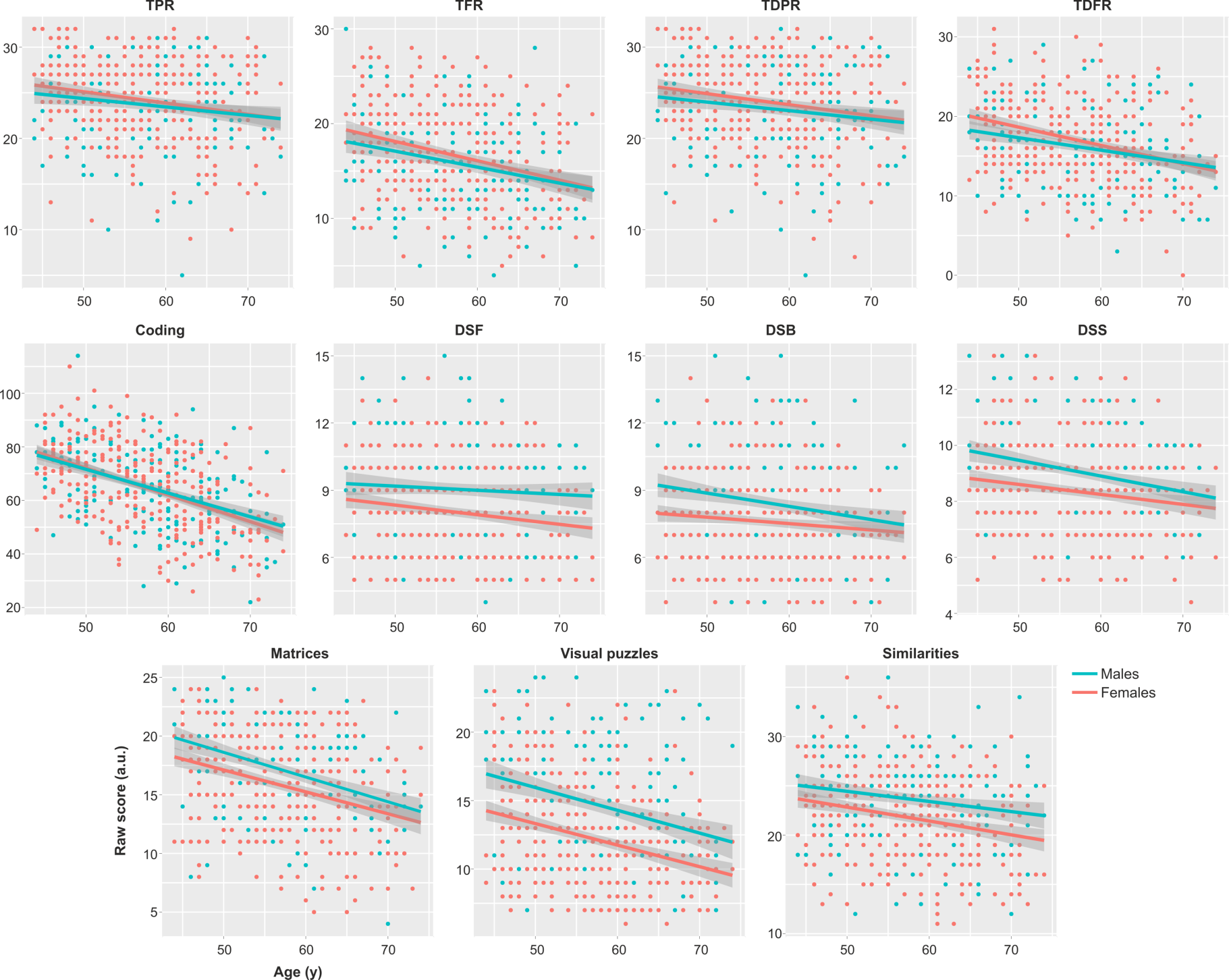


**Supplementary Figure 1.** Age was significantly associated to worse cognitive performance in all outcomes except for the DSF, where results were on a trend level (p = 0.06)
